# Bis{2-methoxy-6-[(4-methyl­phen­yl)iminiometh­yl]­phenolato-κ*O* ^1^}bis­(thio­cyanato-κ*N*)zinc(II)

**DOI:** 10.1107/S1600536808035368

**Published:** 2008-11-08

**Authors:** Hua-Qiong Li, Hui-Duo Xian, Jian-Feng Liu, Guo-Liang Zhao

**Affiliations:** a Zhejiang Key Laboratory for Reactive Chemistry on Solid Surfaces, Institute of Physical Chemistry, Zhejiang Normal University, Jinhua, Zhejiang 321004, People’s Republic of China, and, College of Chemistry and Life Science, Zhejiang Normal University, jinhua 321004, Zhejiang, People’s Republic of China

## Abstract

The Schiff base 2-[(4-methyl­phen­yl)imino­meth­yl]-6-methoxy­phenol (H*L*) forms a complex with a Zn^2+^ atom and two independent thio­cyanate ions, [Zn(NCS)_2_(C_15_H_15_NO_2_)_2_], in which two phenolate O atoms the two independent Schiff base ligands are coordinated to thee Zn^2+^ atom. The protonated imine N atoms are involved in an intramolecular hydrogen bond with the phenoxide group. The Zn atom is also coordinated by two N atoms of two thio­cyanate ligands. The coordination environment of the Zn atom is distorted-tetra­hedral.

## Related literature

For related literature, see: Groeneveld *et al.* (1982 ▶); Iyere *et al.* (2004 ▶); Li (2007 ▶); Maurya *et al.* (1994 ▶); Sen *et al.* (2006 ▶); Yu *et al.* (2007 ▶); Zhang & Wang (2007 ▶); Zhao *et al.* (2007 ▶); Zhou & Zhao (2007 ▶); Zhou *et al.* (2007 ▶). 
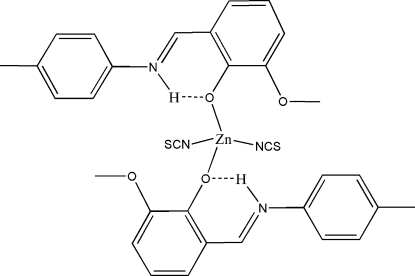


## Experimental

### 

#### Crystal data

[Zn(NCS)_2_(C_15_H_15_NO_2_)_2_]
*M* *_r_* = 664.13
Triclinic, 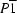

*a* = 9.3830 (2) Å
*b* = 11.7146 (2) Å
*c* = 15.7328 (3) Å
α = 107.7830 (10)°
β = 99.4450 (10)°
γ = 94.1730 (10)°
*V* = 1610.41 (5) Å^3^
*Z* = 2
Mo *K*α radiation
μ = 0.93 mm^−1^
*T* = 296 (2) K
0.23 × 0.18 × 0.07 mm

#### Data collection

Bruker APEXII area-detector diffractometer
Absorption correction: multi-scan (*SADABS*; Sheldrick, 1996 ▶) *T*_min_ = 0.817, *T*_max_ = 0.940
20903 measured reflections
5672 independent reflections
3679 reflections with *I* > 2σ(*I*)
*R*_int_ = 0.034

#### Refinement

*R*[*F* ^2^ > 2σ(*F* ^2^)] = 0.040
*wR*(*F* ^2^) = 0.105
*S* = 1.02
5672 reflections
388 parameters
H-atom parameters constrained
Δρ_max_ = 0.30 e Å^−3^
Δρ_min_ = −0.24 e Å^−3^

### 

Data collection: *APEX2* (Bruker, 2004 ▶); cell refinement: *SAINT* (Bruker, 2004 ▶); data reduction: *SAINT*; program(s) used to solve structure: *SHELXTL* (Sheldrick, 2008 ▶); program(s) used to refine structure: *SHELXTL*; molecular graphics: *SHELXTL*; software used to prepare material for publication: *SHELXTL*.

## Supplementary Material

Crystal structure: contains datablocks I, global. DOI: 10.1107/S1600536808035368/at2651sup1.cif

Structure factors: contains datablocks I. DOI: 10.1107/S1600536808035368/at2651Isup2.hkl

Additional supplementary materials:  crystallographic information; 3D view; checkCIF report

## Figures and Tables

**Table 1:** Hydrogen-bond geometry (Å, °)

*D*—H⋯*A*	*D*—H	H⋯*A*	*D*⋯*A*	*D*—H⋯*A*
N3—H3*A*⋯O2	0.86	1.91	2.594 (3)	135
N4—H4*A*⋯O4	0.86	1.91	2.589 (3)	135

**Fig. 1.**
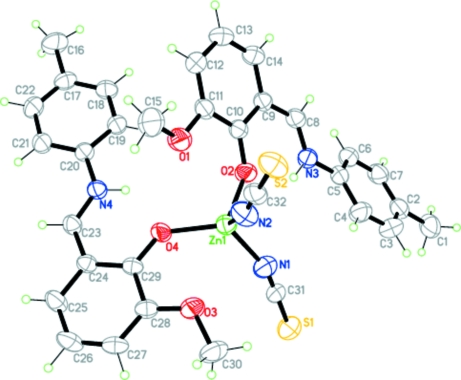
The molecular structure of the title complex, showing the atom-labelling scheme. Displacement ellipsoids are drawn at the 30% probability level.

## supplementary crystallographic information

### Comment

The Schiff base ligands, the products of condensation of *o*-vanillin with amines, viewed several different kind of coordination modes with the centre metals (Sen *et al.*, 2006). And, in early articles, researchers have reported three coordination modes of the Schiff base ligand, HL, derived from condensation of *o*-vanillin and *p*-toluidine. The first mode was that two O atoms of the Schiff base ligand were coordinated to the centre metal (Zhou & Zhao, 2007; Yu *et al.*, 2007; Zhao *et al.*, 2007). The second one was that the ligands coordinate to centre metal through hydroxy O atom and the azomethine N atom (Iyere *et al.*, 2004). The third one was that the centre metal only coordinated to the phenol O atoms (Zhou *et al.*, 2007). In fact, there was still another coordination mode of the Schiff base ligand. It was that all the three donor atoms were coordinated to centre metal, but without its X-ray crystallographic conformations (Maurya *et al.*, 1994). Here we decribe the synthesis and crystal structure of a new zinc(II) complex (Fig. 1), Zn(HL)_2_(NCS)_2_, involing the Schiff base HL, with the third coordination mode.

As shown in Fig. 1, the tridentate ligands coordinate to the Zn atom through two phenolic hydroxy O atoms and two isothiocyanate N atoms, forming a distorted tetrahedral geometry around the metal ion. The isothiocyanate group was not used as a bridged ligand, in the same way to the other Zn complexes with the ligand of isothiocyanate (Li, 2007; Zhang & Wang, 2007; Groeneveld *et al.*, 1982).

### Experimental

The Schiff base was prepared by refluxing *o*-vanillin (10 mmol, 1.5251 g) and *p*-toluidine (10 mmol, 1.0700 g) in ethanol. The colour of the mixture changed from light yellow to orange. Then, for the preparation of the complex, the zinc sulfate heptahydrate (1 mmol, 0.2876 g) and potassium thiocyanate (0.1945 g, 2 mmol) in methanol (10 ml) was added to a methanol (30 ml) solution of the Schiff base ligand (2 mmol, 0.4826 g). Red crystals were obtained after 10 days.

### Refinement

The H atoms bonded to C and N atoms were positioned geometrically and refined using a riding model [aromatic C—H = 0.93 Å, methylic C—H = 0.96 Å, N—H = 0.86 Å, *U*_iso_(H) = 1.2 or 1.5*U*_eq_(C,N)].

### Crystal data

**Table:**

[Zn(NCS)_2_(C_15_H_15_NO_2_)_2_]	*Z* = 2
*M**_r_* = 664.13	*F*_000_ = 688
Triclinic, *P*1	*D*_x_ = 1.370 Mg m^−^^3^
Hall symbol: -P 1	Mo *K*α radiation λ = 0.71073 Å
*a* = 9.3830 (2) Å	Cell parameters from 5295 reflections
*b* = 11.7146 (2) Å	θ = 1.4–25.0º
*c* = 15.7328 (3) Å	µ = 0.93 mm^−^^1^
α = 107.7830 (10)º	*T* = 296 (2) K
β = 99.4450 (10)º	Block, red
γ = 94.1730 (10)º	0.23 × 0.18 × 0.07 mm
*V* = 1610.41 (5) Å^3^	

### Data collection

**Table:**

Bruker APEXII area-detector diffractometer	5672 independent reflections
Radiation source: fine-focus sealed tube	3679 reflections with *I* > 2σ(*I*)
Monochromator: graphite	*R*_int_ = 0.034
*T* = 296(2) K	θ_max_ = 25.0º
ω scans	θ_min_ = 1.4º
Absorption correction: multi-scan(SADABS; Sheldrick, 1996)	*h* = −11→11
*T*_min_ = 0.817, *T*_max_ = 0.940	*k* = −13→13
20903 measured reflections	*l* = −16→18

### Refinement

**Table:**

Refinement on *F*^2^	Secondary atom site location: difference Fourier map
Least-squares matrix: full	Hydrogen site location: inferred from neighbouring sites
*R*[*F*^2^ > 2σ(*F*^2^)] = 0.040	H-atom parameters constrained
*wR*(*F*^2^) = 0.106	*w* = 1/[σ^2^(*F*_o_^2^) + (0.0521*P*)^2^] where *P* = (*F*_o_^2^ + 2*F*_c_^2^)/3
*S* = 1.02	(Δ/σ)_max_ < 0.001
5672 reflections	Δρ_max_ = 0.30 e Å^−^^3^
388 parameters	Δρ_min_ = −0.24 e Å^−^^3^
Primary atom site location: structure-invariant direct methods	Extinction correction: none

### Special details

**Table:**

Geometry. All e.s.d.'s (except the e.s.d. in the dihedral angle between two l.s. planes) are estimated using the full covariance matrix. The cell e.s.d.'s are taken into account individually in the estimation of e.s.d.'s in distances, angles and torsion angles; correlations between e.s.d.'s in cell parameters are only used when they are defined by crystal symmetry. An approximate (isotropic) treatment of cell e.s.d.'s is used for estimating e.s.d.'s involving l.s. planes.
Refinement. Refinement of *F*^2^ against ALL reflections. The weighted *R*-factor *wR* and goodness of fit *S* are based on *F*^2^, conventional *R*-factors *R* are based on *F*, with *F* set to zero for negative *F*^2^. The threshold expression of *F*^2^ > σ(*F*^2^) is used only for calculating *R*-factors(gt) *etc*. and is not relevant to the choice of reflections for refinement. *R*-factors based on *F*^2^ are statistically about twice as large as those based on *F*, and *R*- factors based on ALL data will be even larger.

### Fractional atomic coordinates and isotropic or equivalent isotropic displacement parameters (Å^2^)

**Table:**

	*x*	*y*	*z*	*U*_iso_*/*U*_eq_	
Zn1	−0.34621 (3)	−0.06960 (3)	0.25870 (2)	0.06223 (15)	
S1	−0.56733 (13)	−0.44672 (8)	0.24413 (7)	0.1062 (4)	
O1	−0.3477 (3)	0.1704 (2)	0.25578 (15)	0.0886 (7)	
N1	−0.4233 (3)	−0.2404 (3)	0.2301 (2)	0.0835 (8)	
C1	−1.0051 (5)	−0.6559 (3)	−0.1553 (2)	0.1179 (15)	
H1B	−1.1012	−0.6514	−0.1852	0.177*	
H1C	−1.0118	−0.6975	−0.1118	0.177*	
H1D	−0.9522	−0.6990	−0.1996	0.177*	
S2	0.05039 (9)	0.03726 (9)	0.14475 (6)	0.0846 (3)	
O2	−0.5052 (2)	−0.04420 (18)	0.16903 (13)	0.0691 (6)	
N2	−0.1629 (3)	−0.0426 (3)	0.22310 (19)	0.0825 (8)	
C2	−0.9261 (5)	−0.5293 (3)	−0.1067 (2)	0.0859 (11)	
N3	−0.7024 (2)	−0.1806 (2)	0.03326 (16)	0.0646 (7)	
H3A	−0.6358	−0.1743	0.0797	0.077*	
O3	−0.1914 (2)	−0.1235 (2)	0.39664 (15)	0.0845 (7)	
C3	−0.7856 (5)	−0.5117 (4)	−0.0581 (3)	0.0969 (12)	
H3B	−0.7386	−0.5786	−0.0571	0.116*	
O4	−0.3720 (2)	0.03332 (17)	0.37738 (12)	0.0650 (5)	
N4	−0.5101 (2)	0.2110 (2)	0.44824 (15)	0.0573 (6)	
H4A	−0.4896	0.1612	0.4004	0.069*	
C4	−0.7124 (4)	−0.3966 (3)	−0.0106 (2)	0.0852 (10)	
H4B	−0.6186	−0.3867	0.0232	0.102*	
C5	−0.7802 (3)	−0.2975 (3)	−0.0143 (2)	0.0650 (8)	
C6	−0.9205 (4)	−0.3138 (3)	−0.0617 (2)	0.0771 (9)	
H6A	−0.9679	−0.2472	−0.0631	0.093*	
C7	−0.9912 (4)	−0.4295 (4)	−0.1073 (2)	0.0851 (10)	
H7A	−1.0862	−0.4393	−0.1395	0.102*	
C8	−0.7217 (3)	−0.0826 (3)	0.0137 (2)	0.0647 (8)	
H8A	−0.7944	−0.0879	−0.0357	0.078*	
C9	−0.6408 (3)	0.0308 (3)	0.06162 (19)	0.0576 (8)	
C10	−0.5307 (3)	0.0460 (3)	0.13859 (19)	0.0560 (7)	
C11	−0.4505 (3)	0.1624 (3)	0.18105 (19)	0.0637 (8)	
C12	−0.4809 (4)	0.2564 (3)	0.1503 (2)	0.0739 (9)	
H12A	−0.4268	0.3322	0.1794	0.089*	
C13	−0.5930 (4)	0.2400 (3)	0.0753 (2)	0.0845 (10)	
H13A	−0.6141	0.3051	0.0555	0.101*	
C14	−0.6699 (4)	0.1308 (3)	0.0321 (2)	0.0747 (9)	
H14A	−0.7435	0.1206	−0.0180	0.090*	
C15	−0.2359 (5)	0.2698 (3)	0.2892 (3)	0.1144 (14)	
H15A	−0.1725	0.2635	0.3416	0.172*	
H15B	−0.2783	0.3437	0.3059	0.172*	
H15C	−0.1810	0.2697	0.2428	0.172*	
C16	−0.9135 (4)	0.5296 (3)	0.3767 (3)	0.1022 (12)	
H16A	−0.9594	0.5009	0.3135	0.153*	
H16B	−0.8613	0.6089	0.3911	0.153*	
H16C	−0.9865	0.5333	0.4133	0.153*	
C17	−0.8083 (3)	0.4442 (3)	0.3958 (2)	0.0697 (9)	
C18	−0.7874 (3)	0.3444 (3)	0.3287 (2)	0.0785 (10)	
H18A	−0.8404	0.3271	0.2698	0.094*	
C19	−0.6886 (3)	0.2687 (3)	0.3468 (2)	0.0693 (9)	
H19A	−0.6755	0.2015	0.3002	0.083*	
C20	−0.6098 (3)	0.2925 (3)	0.4334 (2)	0.0553 (7)	
C21	−0.6300 (3)	0.3916 (3)	0.5016 (2)	0.0731 (9)	
H21A	−0.5774	0.4087	0.5605	0.088*	
C22	−0.7291 (4)	0.4658 (3)	0.4820 (2)	0.0789 (10)	
H22A	−0.7428	0.5327	0.5287	0.095*	
C23	−0.4469 (3)	0.2038 (3)	0.52612 (19)	0.0588 (8)	
H23A	−0.4672	0.2573	0.5786	0.071*	
C24	−0.3500 (3)	0.1207 (3)	0.53644 (19)	0.0538 (7)	
C25	−0.2911 (3)	0.1204 (3)	0.6247 (2)	0.0752 (9)	
H25A	−0.3154	0.1754	0.6751	0.090*	
C26	−0.1992 (3)	0.0401 (3)	0.6363 (2)	0.0848 (10)	
H26A	−0.1606	0.0404	0.6947	0.102*	
C27	−0.1621 (3)	−0.0424 (3)	0.5618 (2)	0.0712 (9)	
H27A	−0.0974	−0.0959	0.5712	0.085*	
C28	−0.2183 (3)	−0.0470 (3)	0.4748 (2)	0.0597 (8)	
C29	−0.3157 (3)	0.0361 (2)	0.45939 (19)	0.0517 (7)	
C30	−0.1088 (4)	−0.2196 (3)	0.4021 (3)	0.0940 (11)	
H30A	−0.0982	−0.2661	0.3421	0.141*	
H30B	−0.1583	−0.2708	0.4284	0.141*	
H30C	−0.0142	−0.1865	0.4394	0.141*	
C31	−0.4834 (4)	−0.3275 (3)	0.2352 (2)	0.0683 (8)	
C32	−0.0746 (3)	−0.0081 (3)	0.1911 (2)	0.0637 (8)	

### Atomic displacement parameters (Å^2^)

**Table:**

	*U*^11^	*U*^22^	*U*^33^	*U*^12^	*U*^13^	*U*^23^
Zn1	0.0619 (2)	0.0679 (3)	0.0583 (2)	0.01883 (18)	0.00923 (17)	0.02127 (19)
S1	0.1446 (10)	0.0636 (6)	0.1012 (8)	0.0019 (6)	0.0117 (7)	0.0221 (6)
O1	0.1016 (17)	0.0752 (16)	0.0740 (15)	−0.0010 (14)	−0.0175 (13)	0.0231 (13)
N1	0.091 (2)	0.068 (2)	0.094 (2)	0.0205 (17)	0.0160 (17)	0.0277 (17)
C1	0.181 (4)	0.076 (3)	0.079 (3)	−0.021 (3)	0.019 (3)	0.013 (2)
S2	0.0689 (5)	0.1181 (8)	0.0777 (6)	0.0175 (5)	0.0134 (4)	0.0466 (6)
O2	0.0714 (13)	0.0656 (13)	0.0665 (13)	0.0135 (11)	−0.0055 (10)	0.0249 (11)
N2	0.0642 (19)	0.113 (2)	0.080 (2)	0.0202 (17)	0.0182 (16)	0.0419 (18)
C2	0.120 (3)	0.080 (3)	0.055 (2)	0.003 (3)	0.016 (2)	0.022 (2)
N3	0.0606 (15)	0.0660 (18)	0.0606 (16)	0.0136 (14)	−0.0010 (12)	0.0167 (14)
O3	0.0943 (16)	0.0877 (16)	0.0815 (16)	0.0515 (13)	0.0201 (13)	0.0313 (13)
C3	0.122 (3)	0.071 (3)	0.107 (3)	0.029 (3)	0.024 (3)	0.038 (2)
O4	0.0726 (13)	0.0752 (13)	0.0516 (12)	0.0326 (11)	0.0090 (10)	0.0234 (10)
N4	0.0544 (14)	0.0630 (15)	0.0558 (15)	0.0177 (12)	0.0084 (12)	0.0198 (12)
C4	0.088 (2)	0.076 (3)	0.092 (3)	0.022 (2)	0.007 (2)	0.031 (2)
C5	0.075 (2)	0.067 (2)	0.0516 (18)	0.0148 (18)	0.0102 (16)	0.0172 (17)
C6	0.083 (2)	0.073 (2)	0.066 (2)	0.0131 (19)	−0.0044 (18)	0.0201 (19)
C7	0.090 (3)	0.085 (3)	0.069 (2)	−0.003 (2)	−0.0082 (19)	0.024 (2)
C8	0.0597 (19)	0.078 (2)	0.0592 (19)	0.0197 (18)	0.0087 (15)	0.0245 (18)
C9	0.0566 (18)	0.066 (2)	0.0523 (18)	0.0186 (16)	0.0108 (14)	0.0198 (16)
C10	0.0558 (18)	0.062 (2)	0.0538 (18)	0.0172 (16)	0.0166 (15)	0.0193 (16)
C11	0.070 (2)	0.072 (2)	0.0499 (18)	0.0168 (18)	0.0112 (16)	0.0187 (17)
C12	0.090 (2)	0.061 (2)	0.070 (2)	0.0114 (18)	0.0161 (19)	0.0201 (18)
C13	0.104 (3)	0.078 (3)	0.082 (2)	0.025 (2)	0.008 (2)	0.043 (2)
C14	0.078 (2)	0.078 (2)	0.070 (2)	0.018 (2)	0.0012 (18)	0.032 (2)
C15	0.125 (3)	0.088 (3)	0.098 (3)	−0.015 (3)	−0.027 (3)	0.015 (2)
C16	0.097 (3)	0.097 (3)	0.128 (3)	0.054 (2)	0.022 (2)	0.050 (2)
C17	0.068 (2)	0.064 (2)	0.088 (3)	0.0226 (17)	0.0203 (19)	0.0348 (19)
C18	0.083 (2)	0.081 (2)	0.074 (2)	0.0329 (19)	0.0022 (18)	0.031 (2)
C19	0.079 (2)	0.067 (2)	0.063 (2)	0.0283 (17)	0.0113 (17)	0.0206 (17)
C20	0.0506 (17)	0.0580 (18)	0.0591 (19)	0.0140 (14)	0.0116 (14)	0.0195 (15)
C21	0.085 (2)	0.071 (2)	0.061 (2)	0.0288 (18)	0.0097 (17)	0.0163 (17)
C22	0.090 (2)	0.068 (2)	0.080 (2)	0.0352 (19)	0.024 (2)	0.0155 (19)
C23	0.0541 (17)	0.066 (2)	0.0525 (18)	0.0096 (15)	0.0063 (14)	0.0161 (15)
C24	0.0471 (16)	0.0634 (19)	0.0547 (18)	0.0124 (14)	0.0087 (14)	0.0237 (15)
C25	0.072 (2)	0.100 (3)	0.054 (2)	0.0248 (19)	0.0032 (16)	0.0266 (18)
C26	0.078 (2)	0.117 (3)	0.068 (2)	0.026 (2)	−0.0025 (19)	0.047 (2)
C27	0.060 (2)	0.082 (2)	0.086 (3)	0.0214 (17)	0.0061 (18)	0.049 (2)
C28	0.0475 (17)	0.068 (2)	0.071 (2)	0.0136 (15)	0.0096 (15)	0.0323 (18)
C29	0.0443 (16)	0.0576 (18)	0.0579 (19)	0.0072 (14)	0.0064 (14)	0.0271 (15)
C30	0.096 (3)	0.082 (2)	0.124 (3)	0.048 (2)	0.035 (2)	0.047 (2)
C31	0.084 (2)	0.0578 (18)	0.0596 (19)	0.0249 (14)	0.0083 (17)	0.0139 (17)
C32	0.0567 (19)	0.078 (2)	0.059 (2)	0.0226 (17)	0.0036 (14)	0.0260 (16)

### Geometric parameters (Å, °)

**Table:**

Zn1—N2	1.928 (3)	C10—C11	1.415 (4)
Zn1—O4	1.9531 (19)	C11—C12	1.362 (4)
Zn1—N1	1.965 (3)	C12—C13	1.400 (4)
Zn1—O2	1.9837 (18)	C12—H12A	0.9300
S1—C31	1.609 (4)	C13—C14	1.339 (4)
O1—C11	1.367 (3)	C13—H13A	0.9300
O1—C15	1.414 (4)	C14—H14A	0.9300
N1—C31	1.160 (4)	C15—H15A	0.9600
C1—C2	1.514 (5)	C15—H15B	0.9600
C1—H1B	0.9600	C15—H15C	0.9600
C1—H1C	0.9600	C16—C17	1.516 (4)
C1—H1D	0.9600	C16—H16A	0.9600
S2—C32	1.617 (4)	C16—H16B	0.9600
O2—C10	1.308 (3)	C16—H16C	0.9600
N2—C32	1.147 (4)	C17—C18	1.367 (4)
C2—C7	1.361 (5)	C17—C22	1.373 (4)
C2—C3	1.377 (5)	C18—C19	1.383 (4)
N3—C8	1.293 (3)	C18—H18A	0.9300
N3—C5	1.421 (4)	C19—C20	1.374 (4)
N3—H3A	0.8600	C19—H19A	0.9300
O3—C28	1.357 (3)	C20—C21	1.368 (4)
O3—C30	1.428 (3)	C21—C22	1.380 (4)
C3—C4	1.389 (5)	C21—H21A	0.9300
C3—H3B	0.9300	C22—H22A	0.9300
O4—C29	1.301 (3)	C23—C24	1.406 (4)
N4—C23	1.301 (3)	C23—H23A	0.9300
N4—C20	1.427 (3)	C24—C25	1.409 (4)
N4—H4A	0.8600	C24—C29	1.413 (4)
C4—C5	1.375 (4)	C25—C26	1.353 (4)
C4—H4B	0.9300	C25—H25A	0.9300
C5—C6	1.371 (4)	C26—C27	1.386 (4)
C6—C7	1.382 (4)	C26—H26A	0.9300
C6—H6A	0.9300	C27—C28	1.366 (4)
C7—H7A	0.9300	C27—H27A	0.9300
C8—C9	1.401 (4)	C28—C29	1.430 (4)
C8—H8A	0.9300	C30—H30A	0.9600
C9—C10	1.413 (4)	C30—H30B	0.9600
C9—C14	1.414 (4)	C30—H30C	0.9600
			
N2—Zn1—O4	117.69 (10)	C13—C14—H14A	119.5
N2—Zn1—N1	114.93 (12)	C9—C14—H14A	119.5
O4—Zn1—N1	113.62 (10)	O1—C15—H15A	109.5
N2—Zn1—O2	108.86 (10)	O1—C15—H15B	109.5
O4—Zn1—O2	105.37 (7)	H15A—C15—H15B	109.5
N1—Zn1—O2	92.68 (10)	O1—C15—H15C	109.5
C11—O1—C15	118.8 (3)	H15A—C15—H15C	109.5
C31—N1—Zn1	160.9 (3)	H15B—C15—H15C	109.5
C2—C1—H1B	109.5	C17—C16—H16A	109.5
C2—C1—H1C	109.5	C17—C16—H16B	109.5
H1B—C1—H1C	109.5	H16A—C16—H16B	109.5
C2—C1—H1D	109.5	C17—C16—H16C	109.5
H1B—C1—H1D	109.5	H16A—C16—H16C	109.5
H1C—C1—H1D	109.5	H16B—C16—H16C	109.5
C10—O2—Zn1	133.72 (19)	C18—C17—C22	117.7 (3)
C32—N2—Zn1	161.8 (3)	C18—C17—C16	121.7 (3)
C7—C2—C3	117.6 (3)	C22—C17—C16	120.6 (3)
C7—C2—C1	121.8 (4)	C17—C18—C19	121.0 (3)
C3—C2—C1	120.6 (4)	C17—C18—H18A	119.5
C8—N3—C5	126.5 (3)	C19—C18—H18A	119.5
C8—N3—H3A	116.8	C20—C19—C18	120.3 (3)
C5—N3—H3A	116.8	C20—C19—H19A	119.8
C28—O3—C30	118.5 (2)	C18—C19—H19A	119.8
C2—C3—C4	121.7 (3)	C21—C20—C19	119.5 (3)
C2—C3—H3B	119.2	C21—C20—N4	122.7 (3)
C4—C3—H3B	119.2	C19—C20—N4	117.8 (3)
C29—O4—Zn1	131.10 (17)	C20—C21—C22	119.3 (3)
C23—N4—C20	127.2 (2)	C20—C21—H21A	120.4
C23—N4—H4A	116.4	C22—C21—H21A	120.4
C20—N4—H4A	116.4	C17—C22—C21	122.2 (3)
C5—C4—C3	119.2 (3)	C17—C22—H22A	118.9
C5—C4—H4B	120.4	C21—C22—H22A	118.9
C3—C4—H4B	120.4	N4—C23—C24	124.6 (3)
C6—C5—C4	119.6 (3)	N4—C23—H23A	117.7
C6—C5—N3	122.0 (3)	C24—C23—H23A	117.7
C4—C5—N3	118.4 (3)	C23—C24—C25	119.1 (3)
C5—C6—C7	119.8 (3)	C23—C24—C29	120.6 (2)
C5—C6—H6A	120.1	C25—C24—C29	120.4 (2)
C7—C6—H6A	120.1	C26—C25—C24	120.1 (3)
C2—C7—C6	122.0 (3)	C26—C25—H25A	119.9
C2—C7—H7A	119.0	C24—C25—H25A	119.9
C6—C7—H7A	119.0	C25—C26—C27	120.5 (3)
N3—C8—C9	124.7 (3)	C25—C26—H26A	119.7
N3—C8—H8A	117.7	C27—C26—H26A	119.7
C9—C8—H8A	117.7	C28—C27—C26	121.5 (3)
C8—C9—C10	120.7 (3)	C28—C27—H27A	119.2
C8—C9—C14	119.4 (3)	C26—C27—H27A	119.2
C10—C9—C14	119.9 (3)	O3—C28—C27	127.2 (3)
O2—C10—C9	121.0 (3)	O3—C28—C29	113.0 (3)
O2—C10—C11	121.8 (3)	C27—C28—C29	119.8 (3)
C9—C10—C11	117.2 (3)	O4—C29—C24	121.1 (2)
C12—C11—O1	124.9 (3)	O4—C29—C28	121.3 (3)
C12—C11—C10	121.3 (3)	C24—C29—C28	117.6 (3)
O1—C11—C10	113.8 (3)	O3—C30—H30A	109.5
C11—C12—C13	120.6 (3)	O3—C30—H30B	109.5
C11—C12—H12A	119.7	H30A—C30—H30B	109.5
C13—C12—H12A	119.7	O3—C30—H30C	109.5
C14—C13—C12	120.1 (3)	H30A—C30—H30C	109.5
C14—C13—H13A	119.9	H30B—C30—H30C	109.5
C12—C13—H13A	119.9	N1—C31—S1	178.8 (3)
C13—C14—C9	121.0 (3)	N2—C32—S2	178.7 (3)
			
N2—Zn1—N1—C31	147.9 (8)	O1—C11—C12—C13	−177.1 (3)
O4—Zn1—N1—C31	8.2 (9)	C10—C11—C12—C13	−0.1 (5)
O2—Zn1—N1—C31	−99.8 (8)	C11—C12—C13—C14	−1.1 (5)
N2—Zn1—O2—C10	−55.9 (3)	C12—C13—C14—C9	0.7 (5)
O4—Zn1—O2—C10	71.2 (3)	C8—C9—C14—C13	−178.8 (3)
N1—Zn1—O2—C10	−173.4 (3)	C10—C9—C14—C13	1.0 (5)
O4—Zn1—N2—C32	−94.6 (10)	C22—C17—C18—C19	−0.7 (5)
N1—Zn1—N2—C32	127.4 (10)	C16—C17—C18—C19	178.5 (3)
O2—Zn1—N2—C32	25.1 (10)	C17—C18—C19—C20	0.3 (5)
C7—C2—C3—C4	0.6 (5)	C18—C19—C20—C21	0.1 (5)
C1—C2—C3—C4	−178.0 (3)	C18—C19—C20—N4	179.7 (3)
N2—Zn1—O4—C29	−72.2 (3)	C23—N4—C20—C21	13.6 (5)
N1—Zn1—O4—C29	66.3 (3)	C23—N4—C20—C19	−166.0 (3)
O2—Zn1—O4—C29	166.2 (2)	C19—C20—C21—C22	0.0 (5)
C2—C3—C4—C5	−1.9 (6)	N4—C20—C21—C22	−179.6 (3)
C3—C4—C5—C6	2.5 (5)	C18—C17—C22—C21	0.7 (5)
C3—C4—C5—N3	−178.7 (3)	C16—C17—C22—C21	−178.5 (3)
C8—N3—C5—C6	−27.7 (5)	C20—C21—C22—C17	−0.4 (5)
C8—N3—C5—C4	153.5 (3)	C20—N4—C23—C24	179.5 (3)
C4—C5—C6—C7	−1.7 (5)	N4—C23—C24—C25	−178.6 (3)
N3—C5—C6—C7	179.6 (3)	N4—C23—C24—C29	−0.7 (4)
C3—C2—C7—C6	0.2 (5)	C23—C24—C25—C26	179.1 (3)
C1—C2—C7—C6	178.8 (3)	C29—C24—C25—C26	1.1 (5)
C5—C6—C7—C2	0.3 (5)	C24—C25—C26—C27	0.0 (5)
C5—N3—C8—C9	−179.0 (3)	C25—C26—C27—C28	−1.1 (5)
N3—C8—C9—C10	−1.4 (5)	C30—O3—C28—C27	7.6 (5)
N3—C8—C9—C14	178.4 (3)	C30—O3—C28—C29	−173.2 (3)
Zn1—O2—C10—C9	168.24 (19)	C26—C27—C28—O3	−179.6 (3)
Zn1—O2—C10—C11	−12.0 (4)	C26—C27—C28—C29	1.1 (5)
C8—C9—C10—O2	−2.6 (4)	Zn1—O4—C29—C24	179.42 (18)
C14—C9—C10—O2	177.6 (3)	Zn1—O4—C29—C28	−1.2 (4)
C8—C9—C10—C11	177.7 (3)	C23—C24—C29—O4	0.4 (4)
C14—C9—C10—C11	−2.1 (4)	C25—C24—C29—O4	178.3 (3)
C15—O1—C11—C12	−19.6 (5)	C23—C24—C29—C28	−179.0 (3)
C15—O1—C11—C10	163.2 (3)	C25—C24—C29—C28	−1.1 (4)
O2—C10—C11—C12	−178.0 (3)	O3—C28—C29—O4	1.2 (4)
C9—C10—C11—C12	1.7 (4)	C27—C28—C29—O4	−179.4 (3)
O2—C10—C11—O1	−0.7 (4)	O3—C28—C29—C24	−179.4 (2)
C9—C10—C11—O1	179.0 (2)	C27—C28—C29—C24	0.0 (4)

### Hydrogen-bond geometry (Å, °)

**Table:**

*D*—H···*A*	*D*—H	H···*A*	*D*···*A*	*D*—H···*A*
N3—H3A···O2	0.86	1.91	2.594 (3)	135
N4—H4A···O4	0.86	1.91	2.589 (3)	135
